# Crystal structure of poly[[[μ_4_-3-(1,2,4-triazol-4-yl)adamantane-1-carboxyl­ato-κ^5^ *N* ^1^:*N* ^2^:*O* ^1^:*O* ^1^,*O* ^1′^]silver(I)] dihydrate]

**DOI:** 10.1107/S2056989019009708

**Published:** 2019-07-12

**Authors:** Ganna A. Senchyk, Harald Krautscheid, Kostiantyn V. Domasevitch

**Affiliations:** a Inorganic Chemistry Department, Taras Shevchenko National University of Kyiv, Volodymyrska Street, 64, Kyiv 01033, Ukraine; b Institut für Anorganische Chemie, Universitat Leipzig, Johannisallee 29, D-04103, Leipzig, Germany

**Keywords:** 1,2,4-triazolyl carboxyl­ate, silver(I) metal-organic framework, hydrogen bonding, crystal structure

## Abstract

The heterobifunctional organic ligand, 3-(1,2,4-triazol-4-yl)adamantane-1-carboxyl­ate (*tr-ad-COO^−^*), was employed for the synthesis of the title silver(I) coordination polymer, {[Ag(C_13_H_16_N_3_O_2_)]·2H_2_O}_*n*_, crystallizing in the rare ortho­rhom­bic *C*222_1_ space group. Alternation of the double μ_2_-1,2,4-triazole and μ_2_-η^2^:η^1^-COO^−^ (chelating, bridging mode) bridges between Ag^I^ cations supports the formation of sinusoidal coordination chains. The Ag^I^ centers possess a distorted {N_2_O_3_} square-pyramidal arrangement with τ_5_ = 0.30. The angular organic linkers connect the chains into a tetra­gonal framework with small channels along the *c-*axis direction occupied by water mol­ecules of crystallization, which are inter­linked *via* O—H⋯O hydrogen bonds with carboxyl­ate groups, leading to right- and left-handed helical dispositions.

## Abstract 2

The title Ag^I^ compound based on 3-(1,2,4-triazol-4-yl)adamantane-1-carboxyl­ate (*tr-ad-COO^−^*) is a three-dimensional coordination framework with channels along the *c*-axis direction. Hydrogen bonding between water mol­ecules of crystallization and carboxyl­ate groups is realised in form of right- and left-handed helical motifs.

## Chemical context   

Organic ligands, which contain two different functional groups, such as azole and carb­oxy­lic groups, attract attention in the context of the construction of unusual metal–organic frameworks (MOFs) including heterometallic architectures (Guillerm *et al.* 2014 ▸). Each ligand function is intended to introduce its coordination ability towards a metal center forming secondary building units (SBUs) based on its peculiarities. For instance, 1,2,4-triazoles (*tr*) typically serve as short *N*,*N*-bridges between two metal ions resulting in polynuclear units and chains (Wang *et al.* 2007 ▸, Murdock & Jenkins 2014 ▸). In contrast, carboxyl­ate groups offer a much broader variety of coordination modes: mono-, chelate-, bridging- and their combinations; and the number of connected metal ions may differ from one to four (Sun *et al.* 2004 ▸; Lu *et al.* 2014 ▸). As shown by Lincke *et al.* (2011 ▸, 2012 ▸), 1,2,4-triazole­carboxyl­ate ligands are good candidates for the construction of microporous MOFs suitable for gas sorption and separation. Considering the heterofunctional *tr/COO* ligands, there are two possible roles for them to play. First, the ‘separate’ role, where *tr* is responsible for di-, tri- or tetra­nuclear cluster formation, whereas the *COO^−^* group only occupies terminal (non-bridging) positions (Handke *et al.* 2014 ▸) or it can be involved in the separate coordination to metal centers. In this context, Chen *et al.* (2011 ▸) used 1,2,4-triazolyl isophthalate as a ligand in the synthesis of a series of Ag^I^–*Ln* ^III^ heterometallic coordination polymers. Second, in the ‘cooperative’ role, *tr/COO* serves as a heteroleptic bridge between the metal centers (Vasylevs’kyy *et al.* 2015 ▸).
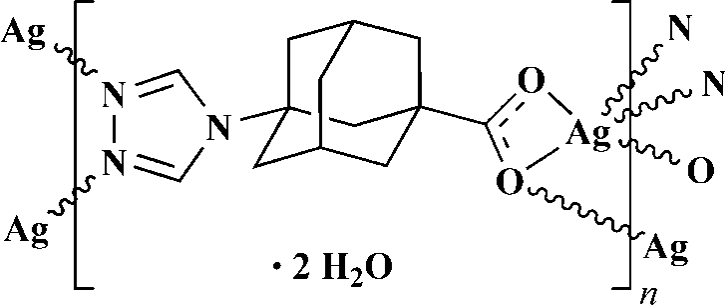


In present paper, we report the crystal structure of a new silver(I) coordination polymer [Ag(*tr-ad-COO*)]_*n*_·2H_2_O (**I**) based on the 1-(1,2,4-triazol-4-yl)-3-carb­oxy­adamantane (C_13_H_16_N_3_O_2_; *tr-ad-COOH*) ligand.

## Structural commentary   

**Figure 1:**
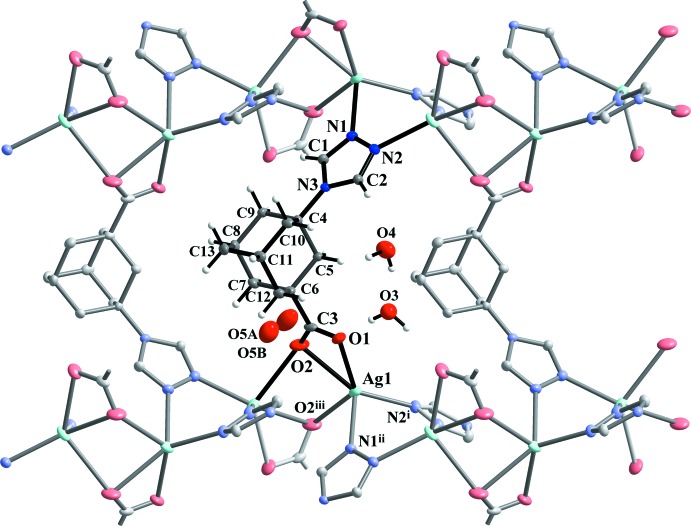
Fragment of the crystal structure of **I**. The independent part of the structure is indicated with black bonds and displacement ellipsoids are drawn at the 50% probability level. [Symmetry codes: (i) 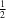 − *x*, −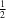 + *y*, 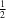 − *z*, (ii) 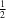 + *x*, −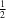 + *y*, *z*, (iii) *x*, −*y*, 1 − *z*]

The title compound **I** crystallizes in the ortho­rhom­bic system with the uncommon space group *C222_1_*. The asymmetric unit contains one Ag^I^ cation, one organic ligand and three distinct water mol­ecules of crystallization, one of which (O5) is disordered over two adjacent sites (Fig. 1 ▸). The O3 water mol­ecule is situated on a crystallographic twofold axis passing through the O atom, while the O4 water molecule is statistically disordered over two positions, both possessing an occupancy factor of 0.5. Thus, in the asymmetric unit, the total atom content sums up to two water molecules. The 1,2,4-triazole functional group is coordinated by two Ag^I^ centers as a μ_2_-*N*,*N* bridge and the carboxyl­ate group connects two Ag^I^ centers in a chelating, bridging mode (μ_2_-η^2^:η^1^), supporting the formation of sinusoidal chains with a periodicity of 13 Å.

**Figure 2:**
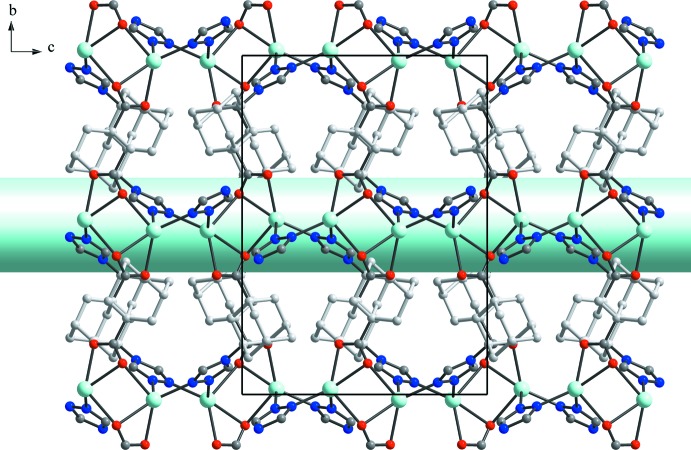
Projection on the *bc* plane showing the inter­connection of sinusoidal Ag^I^ coordination chains by means of *tr-ad-COO^−^* organic ligands into a three-dimensional framework.

**Figure 3:**
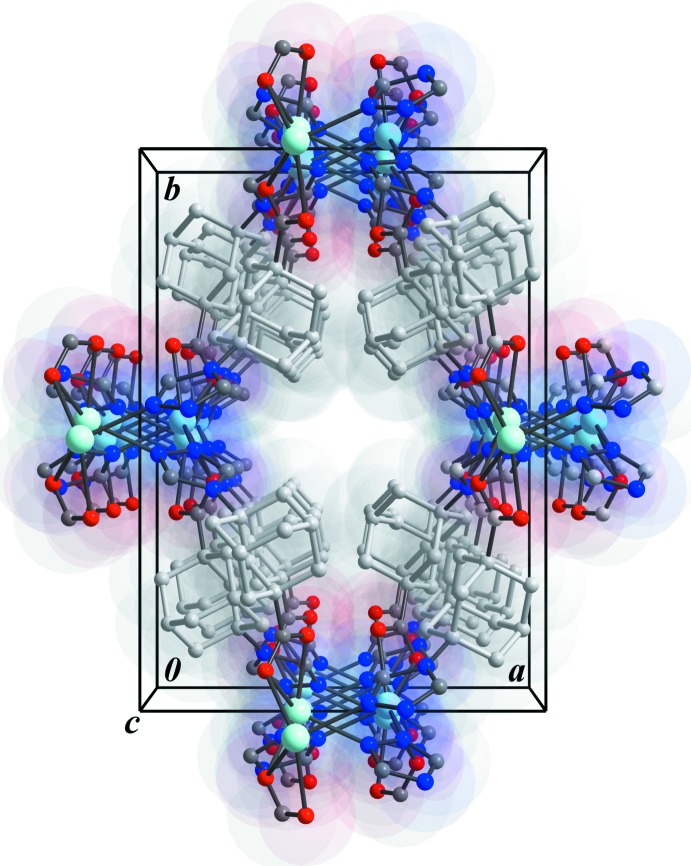
View of the channels along the *c* axis in the structure of **I**.

In the case of compound **I** an unusual situation with alternation of double triazoles and double carboxyl­ate bridges within the chain is observed. Thus, the *tr-ad-COO^−^* ligands act in a deprotonated form adopting a μ_4_-coordination modes (Fig. 2 ▸) that yields a three-dimensional tetra­gonal pattern with open channels along the *c-*axis direction (Fig. 3 ▸).

The coordination environment of the Ag^I^ cation is a very distorted {N_2_O_3_} polyhedron with two Ag—N(triazole) [2.291 (3) Å, 2.442 (3) Å] and three elongated Ag—O(carboxyl­ate) [2.437 (3)–2.703 (4) Å] bonds (Table 1 ▸). The geometry of the five-coordinate center can be described by the geometric parameter τ_5_, which represents the degree of trigonality between two ideal structures – trigonal bipyramid (τ_5_ = 1) and square pyramid (τ_5_ = 0) (Addison *et al.*, 1984 ▸). In compound **I**, the Ag1 center has τ_5_ = 0.30, indicating a significantly distorted square-pyramidal geometry.

## Supra­molecular features   

**Figure 4:**
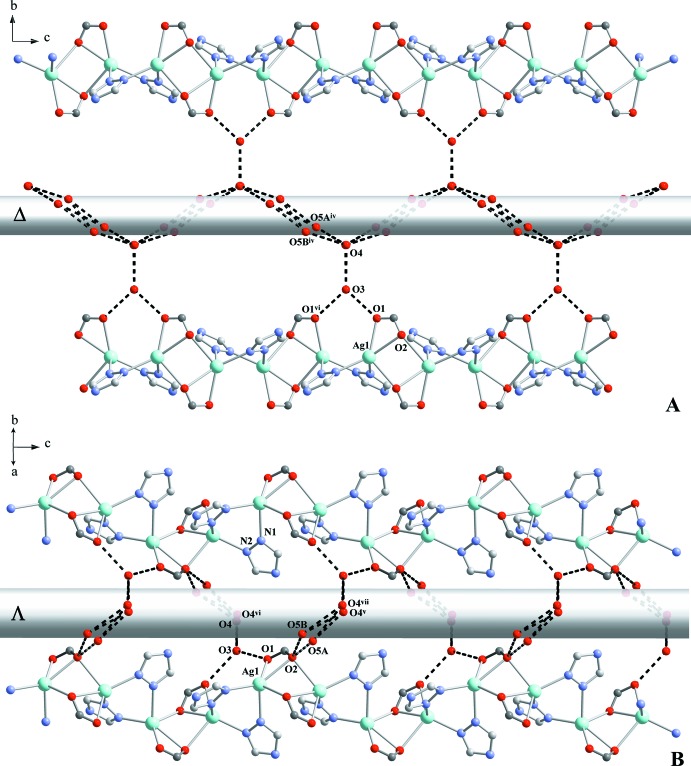
Hydrogen-bonding scheme between water mol­ecules and COO^−^ groups showing the formation of right-handed (A) and left-handed (B) helices. Adamantyl fragments are omitted for clarity. [Symmetry codes: (iv) 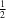 + *x*, 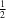 − *y*, 1 − *z*; (v) − 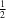 + *x*, 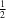 − *y*, 1 − *z*; (vi) 1 − *x*, *y*, 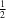 − *z*; (vii) 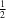 − *x*, 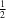 − *y*, 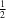 + *z.*]

**Figure 5:**
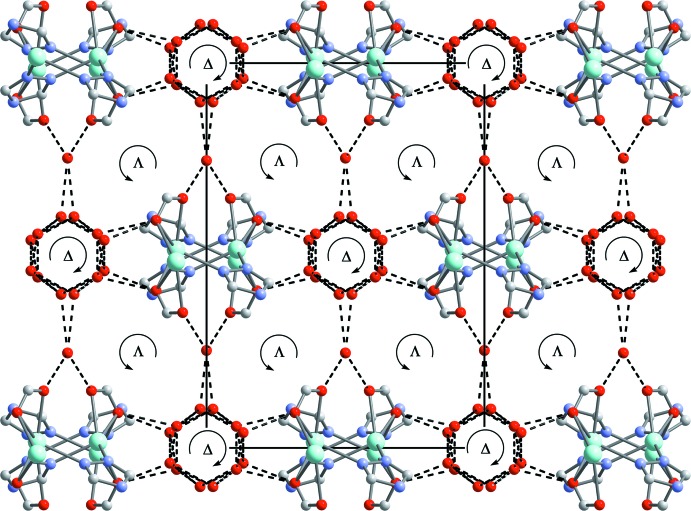
The packing of the right- and left-handed helices in the crystal structure of **I** (top view). Adamantyl fragments are omitted for clarity.

The water guest mol­ecules inside the [001] channels are responsible for the extended hydrogen-bonding network (Table 2 ▸). Together with the –COO^−^ groups, they are organized into two types of helices along the *c* axis – smaller right-handed (A in Fig. 4 ▸) and bigger left-handed (B in Fig. 4 ▸). In addition, weak C—H_(triazole)_⋯O1_(COO)_ and C—H_(triazole)_⋯O4_(water)_ contacts are observed. The packig is shown in Fig. 5 ▸.

## Synthesis and crystallization   

1-(1,2,4-Triazol-4-yl)-3-carb­oxy­adamantane (*tr-ad-COOH*) was synthesized by refluxing 3-amino-adamantane-1-carb­oxy­lic acid (Wanka *et al.*, 2007 ▸) (3.00 g, 15.4 mmol) and di­methyl­formamide azine (5.46 g, 38.5 mmol) in the presence of toluene­sulfonic acid monohydrate (0.44 g, 2.3 mmol) as catalyst in DMF (30 ml). Yield = 63%.

The synthesis of **I** was carried out under hydro­thermal conditions as follows. A mixture of AgNO_3_ (17.0 mg, 0.100 mmol), *tr-ad-COOH* (12.4 mg, 0.050 mmol) and 5 ml of water was added into a Teflon vessel, which was sealed and heated at 413 K for 24 h and slowly cooled to room temperature over 48 h, yielding colourless needles of **I** (yield 13.3 mg, 68%).

## Refinement   

Crystal data, data collection and structure refinement details are summarized in Table 3 ▸. O4 lies adjacent to a crystallographic twofold axis and is statistically disordered over two positions (O4⋯O4 = 0.60 Å) and O5 is statistically disordered over adjacent locations (O5*A*⋯O5*B* = 0.77 Å). CH hydrogen atoms were positioned geometrically and refined as riding, with C—H = 0.94 Å (triazole); C—H = 0.98 Å (adamantane CH_2_); C—H = 0.99 Å (adamantane CH) and with *U*_iso_(H) = 1.2*U*_eq_(C). OH hydrogen atoms were located and then refined with O—H = 0.85 Å (H_2_O) and with *U*_iso_(H) = 1.5*U*_eq_(O). For one of the disordered water mol­ecules, the H atoms were not located.

## Supplementary Material

Crystal structure: contains datablock(s) I. DOI: 10.1107/S2056989019009708/hb7837sup1.cif

Structure factors: contains datablock(s) I. DOI: 10.1107/S2056989019009708/hb7837Isup2.hkl

CCDC reference: 1939097

Additional supporting information:  crystallographic information; 3D view; checkCIF report

## Figures and Tables

**Table 1:** Selected geometric parameters (Å, °)

Ag1—N2^i^	2.291 (3)	Ag1—O2	2.571 (4)
Ag1—O1	2.437 (3)	Ag1—O2^iii^	2.703 (4)
Ag1—N1^ii^	2.442 (3)		
			
N2^i^—Ag1—O1	125.93 (11)	N1^ii^—Ag1—O2	121.93 (12)
N2^i^—Ag1—N1^ii^	92.12 (11)	N2^i^—Ag1—O2^iii^	101.35 (11)
O1—Ag1—N1^ii^	106.84 (11)	O1—Ag1—O2^iii^	128.04 (11)
N2^i^—Ag1—O2	145.74 (12)	N1^ii^—Ag1—O2^iii^	89.79 (11)
O1—Ag1—O2	51.95 (11)	O2—Ag1—O2^iii^	77.22 (13)

Symmetry codes: (i) 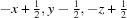; (ii) 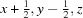; (iii) 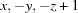.

**Table 2:** Hydrogen-bond geometry (Å, °)

*D*—H⋯*A*	*D*—H	H⋯*A*	*D*⋯*A*	*D*—H⋯*A*
O3—H1*W*⋯O1	0.85	1.97	2.818 (5)	171
O4—H2*W*⋯O3	0.85	1.92	2.746 (10)	163
O4—H3*W*⋯O5*A* ^iv^	0.85	1.81	2.56 (2)	147
O4—H3*W*⋯O5*B* ^iv^	0.85	2.11	2.83 (3)	143
C1—H1⋯O1^v^	0.94	2.43	3.336 (5)	162
C2—H2⋯O4^vi^	0.94	2.58	3.516 (12)	171

Symmetry codes: (iv) 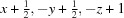; (v) 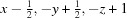; (vi) 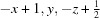.

**Table 3:** Experimental details

Crystal data	
Chemical formula	[Ag(C_13_H_16_N_3_O_2_)]·2H_2_O
*M*_r_	390.19
Crystal system, space group	Orthorhombic, *C*222_1_
Temperature (K)	213
*a*, *b*, *c* (Å)	12.9321 (9), 17.9056 (10), 12.9695 (9)
*V* (Å^3^)	3003.2 (3)
*Z*	8
Radiation type	Mo *K*α
μ (mm^−1^)	1.36
Crystal size (mm)	0.22 × 0.18 × 0.16

Data collection	
Diffractometer	Stoe Image plate diffraction system
Absorption correction	Numerical [*X-RED* (Stoe & Cie, 2001 ▸) and *X-SHAPE* (Stoe & Cie, 1999 ▸)]
*T*_min_, *T*_max_	0.649, 0.689
No. of measured, independent and observed [*I* > 2σ(*I*)] reflections	13596, 3617, 3135
*R*_int_	0.028
(sin θ/λ)_max_ (Å^−1^)	0.661

Refinement	
*R*[*F* ^2^ > 2σ(*F* ^2^)], *wR*(*F* ^2^), *S*	0.025, 0.058, 0.97
No. of reflections	3617
No. of parameters	204
H-atom treatment	H-atom parameters constrained
Δρ_max_, Δρ_min_ (e Å^−3^)	0.89, −0.94
Absolute structure	Flack *x* determined using 1289 quotients [(*I* ^+^)−(*I* ^−^)]/[(*I* ^+^)+(*I* ^−^)] (Parsons et al., 2013 ▸)
Absolute structure parameter	−0.060 (9)

Computer programs: *IPDS Software* (Stoe & Cie, 2000 ▸), *SHELXS97* (Sheldrick, 2008 ▸), *SHELXL2014* (Sheldrick, 2015 ▸), *DIAMOND* (Brandenburg, 1999 ▸) and *WinGX* (Farrugia, 2012 ▸).

## supplementary crystallographic information

### Crystal data

**Table:**

[Ag(C_13_H_16_N_3_O_2_)]·2H_2_O	*D*_x_ = 1.726 Mg m^−^^3^
*M**_r_* = 390.19	Mo *K*α radiation, λ = 0.71073 Å
Orthorhombic, *C*222_1_	Cell parameters from 13596 reflections
*a* = 12.9321 (9) Å	θ = 2.8–28.0°
*b* = 17.9056 (10) Å	µ = 1.36 mm^−^^1^
*c* = 12.9695 (9) Å	*T* = 213 K
*V* = 3003.2 (3) Å^3^	Needle, colourless
*Z* = 8	0.22 × 0.18 × 0.16 mm
*F*(000) = 1584	

### Data collection

**Table:**

Stoe Image plate diffraction system diffractometer	3135 reflections with *I* > 2σ(*I*)
φ oscillation scans	*R*_int_ = 0.028
Absorption correction: numerical [X-RED (Stoe & Cie, 2001) and X-SHAPE (Stoe & Cie, 1999)]	θ_max_ = 28.0°, θ_min_ = 2.8°
*T*_min_ = 0.649, *T*_max_ = 0.689	*h* = −17→17
13596 measured reflections	*k* = −22→23
3617 independent reflections	*l* = −17→17

### Refinement

**Table:**

Refinement on *F*^2^	Hydrogen site location: mixed
Least-squares matrix: full	H-atom parameters constrained
*R*[*F*^2^ > 2σ(*F*^2^)] = 0.025	*w* = 1/[σ^2^(*F*_o_^2^) + (0.037*P*)^2^] where *P* = (*F*_o_^2^ + 2*F*_c_^2^)/3
*wR*(*F*^2^) = 0.058	(Δ/σ)_max_ = 0.001
*S* = 0.97	Δρ_max_ = 0.89 e Å^−^^3^
3617 reflections	Δρ_min_ = −0.94 e Å^−^^3^
204 parameters	Absolute structure: Flack *x* determined using 1289 quotients [(*I*^+^)-(*I*^-^)]/[(*I*^+^)+(*I*^-^)] (Parsons et al., 2013)
0 restraints	Absolute structure parameter: −0.060 (9)

### Special details

**Table:**

Geometry. All esds (except the esd in the dihedral angle between two l.s. planes) are estimated using the full covariance matrix. The cell esds are taken into account individually in the estimation of esds in distances, angles and torsion angles; correlations between esds in cell parameters are only used when they are defined by crystal symmetry. An approximate (isotropic) treatment of cell esds is used for estimating esds involving l.s. planes.

### Fractional atomic coordinates and isotropic or equivalent isotropic displacement parameters (Å^2^)

**Table:**

	*x*	*y*	*z*	*U*_iso_*/*U*_eq_	Occ. (<1)
Ag1	0.39032 (3)	0.01581 (2)	0.35981 (2)	0.04198 (10)	
O1	0.4141 (3)	0.14822 (17)	0.3962 (2)	0.0474 (8)	
O2	0.3185 (3)	0.09152 (19)	0.5123 (3)	0.0581 (9)	
N1	0.0616 (2)	0.45845 (17)	0.3648 (3)	0.0318 (6)	
N2	0.1451 (2)	0.46474 (17)	0.2982 (2)	0.0316 (7)	
N3	0.1946 (2)	0.39903 (16)	0.4325 (2)	0.0231 (6)	
C1	0.0930 (3)	0.4190 (2)	0.4435 (3)	0.0282 (8)	
H1	0.0514	0.4060	0.5001	0.034*	
C2	0.2230 (3)	0.4290 (2)	0.3398 (2)	0.0276 (7)	
H2	0.2890	0.4245	0.3102	0.033*	
C3	0.3583 (3)	0.1495 (2)	0.4758 (3)	0.0339 (9)	
C4	0.2574 (2)	0.3521 (2)	0.5055 (3)	0.0219 (7)	
C5	0.2762 (3)	0.27539 (19)	0.4550 (3)	0.0234 (7)	
H5A	0.3139	0.2819	0.3900	0.028*	
H5B	0.2098	0.2515	0.4396	0.028*	
C6	0.3398 (3)	0.2253 (2)	0.5293 (3)	0.0260 (7)	
C7	0.2807 (3)	0.2174 (2)	0.6319 (3)	0.0333 (8)	
H7A	0.2140	0.1929	0.6196	0.040*	
H7B	0.3206	0.1862	0.6796	0.040*	
C8	0.2626 (3)	0.2948 (2)	0.6802 (3)	0.0333 (9)	
H8	0.2247	0.2887	0.7460	0.040*	
C9	0.1977 (3)	0.3427 (2)	0.6064 (3)	0.0278 (8)	
H9A	0.1311	0.3184	0.5931	0.033*	
H9B	0.1843	0.3917	0.6374	0.033*	
C10	0.3616 (3)	0.3907 (2)	0.5253 (3)	0.0287 (8)	
H10A	0.3502	0.4401	0.5556	0.034*	
H10B	0.3990	0.3970	0.4601	0.034*	
C11	0.4256 (3)	0.3416 (2)	0.5999 (3)	0.0326 (8)	
H11	0.4930	0.3659	0.6135	0.039*	
C12	0.4439 (3)	0.2646 (2)	0.5505 (3)	0.0294 (8)	
H12A	0.4859	0.2338	0.5968	0.035*	
H12B	0.4819	0.2707	0.4857	0.035*	
C13	0.3663 (3)	0.3327 (2)	0.7016 (3)	0.0384 (10)	
H13A	0.4069	0.3025	0.7499	0.046*	
H13B	0.3547	0.3818	0.7328	0.046*	
O3	0.5000	0.2468 (3)	0.2500	0.0630 (13)	
H1W	0.4787	0.2190	0.2988	0.094*	
O4	0.5231 (6)	0.3992 (4)	0.2479 (18)	0.068 (3)	0.5
H2W	0.5153	0.3530	0.2348	0.102*	0.5
H3W	0.5724	0.4038	0.2909	0.102*	0.5
O5A	0.1318 (13)	0.0398 (10)	0.6097 (11)	0.096 (4)	0.5
O5B	0.1073 (12)	0.0560 (11)	0.5604 (12)	0.100 (5)	0.5

### Atomic displacement parameters (Å^2^)

**Table:**

	*U*^11^	*U*^22^	*U*^33^	*U*^12^	*U*^13^	*U*^23^
Ag1	0.04963 (17)	0.03609 (16)	0.04023 (15)	0.01122 (15)	−0.00959 (15)	−0.01749 (13)
O1	0.063 (2)	0.0348 (16)	0.0446 (17)	0.0145 (14)	−0.0012 (14)	−0.0141 (12)
O2	0.064 (2)	0.0231 (18)	0.087 (3)	0.0013 (17)	0.005 (2)	−0.0047 (15)
N1	0.0325 (15)	0.0318 (15)	0.0311 (14)	0.0098 (12)	−0.0019 (15)	−0.0012 (14)
N2	0.0379 (17)	0.0292 (17)	0.0275 (14)	0.0059 (13)	−0.0028 (12)	0.0018 (12)
N3	0.0255 (15)	0.0201 (15)	0.0239 (14)	0.0042 (12)	−0.0023 (11)	0.0004 (11)
C1	0.0258 (19)	0.0321 (19)	0.0266 (17)	0.0057 (15)	0.0039 (14)	0.0016 (13)
C2	0.0290 (17)	0.0307 (19)	0.0231 (17)	0.0050 (15)	0.0002 (13)	0.0037 (13)
C3	0.037 (2)	0.019 (2)	0.045 (2)	0.0076 (16)	−0.0134 (16)	−0.0061 (17)
C4	0.0260 (15)	0.022 (2)	0.0176 (13)	0.0035 (15)	−0.0030 (14)	0.0009 (11)
C5	0.0261 (16)	0.0211 (17)	0.0229 (15)	0.0034 (14)	−0.0023 (13)	−0.0028 (13)
C6	0.0317 (18)	0.0184 (17)	0.0280 (17)	0.0040 (15)	−0.0026 (13)	−0.0024 (13)
C7	0.0394 (18)	0.0290 (19)	0.0315 (18)	0.0046 (15)	0.0011 (17)	0.0080 (16)
C8	0.042 (2)	0.039 (2)	0.0196 (15)	0.0089 (17)	0.0015 (15)	0.0045 (14)
C9	0.0321 (19)	0.0278 (19)	0.0235 (16)	0.0076 (15)	0.0039 (13)	0.0010 (13)
C10	0.0305 (19)	0.0208 (19)	0.0349 (19)	−0.0023 (14)	−0.0063 (13)	−0.0024 (15)
C11	0.0323 (19)	0.0275 (19)	0.0380 (19)	0.0011 (15)	−0.0147 (15)	−0.0050 (15)
C12	0.0272 (18)	0.029 (2)	0.0321 (19)	0.0056 (16)	−0.0059 (14)	−0.0003 (15)
C13	0.052 (3)	0.037 (2)	0.0266 (17)	0.0087 (18)	−0.0121 (16)	−0.0052 (15)
O3	0.067 (3)	0.060 (3)	0.062 (3)	0.000	0.001 (3)	0.000
O4	0.051 (9)	0.068 (4)	0.085 (5)	−0.008 (4)	−0.018 (10)	0.000 (6)
O5A	0.089 (10)	0.109 (9)	0.091 (10)	−0.005 (7)	0.009 (7)	−0.018 (8)
O5B	0.060 (7)	0.128 (12)	0.110 (11)	0.003 (8)	−0.005 (8)	−0.045 (10)

### Geometric parameters (Å, º)

**Table:**

Ag1—N2^i^	2.291 (3)	C6—C12	1.544 (5)
Ag1—O1	2.437 (3)	C7—C8	1.538 (5)
Ag1—N1^ii^	2.442 (3)	C7—H7A	0.9800
Ag1—O2	2.571 (4)	C7—H7B	0.9800
Ag1—O2^iii^	2.703 (4)	C8—C13	1.529 (6)
O1—C3	1.259 (5)	C8—C9	1.536 (5)
O2—C3	1.251 (5)	C8—H8	0.9900
N1—C1	1.305 (5)	C9—H9A	0.9800
N1—N2	1.387 (4)	C9—H9B	0.9800
N1—Ag1^iv^	2.442 (3)	C10—C11	1.548 (5)
N2—C2	1.310 (5)	C10—H10A	0.9800
N2—Ag1^v^	2.291 (3)	C10—H10B	0.9800
N3—C2	1.366 (4)	C11—C13	1.533 (6)
N3—C1	1.369 (5)	C11—C12	1.538 (5)
N3—C4	1.505 (4)	C11—H11	0.9900
C1—H1	0.9400	C12—H12A	0.9800
C2—H2	0.9400	C12—H12B	0.9800
C3—C6	1.544 (5)	C13—H13A	0.9800
C4—C9	1.530 (5)	C13—H13B	0.9800
C4—C10	1.535 (5)	O3—H1W	0.8500
C4—C5	1.541 (5)	O4—O4^vi^	0.601 (16)
C5—C6	1.552 (5)	O4—H2W	0.8500
C5—H5A	0.9800	O4—H3W	0.8500
C5—H5B	0.9800	O5A—O5B	0.770 (16)
C6—C7	1.542 (5)		
			
N2^i^—Ag1—O1	125.93 (11)	C3—C6—C5	108.1 (3)
N2^i^—Ag1—N1^ii^	92.12 (11)	C8—C7—C6	110.1 (3)
O1—Ag1—N1^ii^	106.84 (11)	C8—C7—H7A	109.6
N2^i^—Ag1—O2	145.74 (12)	C6—C7—H7A	109.6
O1—Ag1—O2	51.95 (11)	C8—C7—H7B	109.6
N1^ii^—Ag1—O2	121.93 (12)	C6—C7—H7B	109.6
N2^i^—Ag1—O2^iii^	101.35 (11)	H7A—C7—H7B	108.2
O1—Ag1—O2^iii^	128.04 (11)	C13—C8—C9	110.1 (3)
N1^ii^—Ag1—O2^iii^	89.79 (11)	C13—C8—C7	110.0 (3)
O2—Ag1—O2^iii^	77.22 (13)	C9—C8—C7	109.5 (3)
C3—O1—Ag1	96.0 (3)	C13—C8—H8	109.1
C3—O2—Ag1	89.9 (3)	C9—C8—H8	109.1
C1—N1—N2	106.7 (3)	C7—C8—H8	109.1
C1—N1—Ag1^iv^	122.0 (2)	C4—C9—C8	108.5 (3)
N2—N1—Ag1^iv^	130.9 (2)	C4—C9—H9A	110.0
C2—N2—N1	107.6 (3)	C8—C9—H9A	110.0
C2—N2—Ag1^v^	135.8 (2)	C4—C9—H9B	110.0
N1—N2—Ag1^v^	115.7 (2)	C8—C9—H9B	110.0
C2—N3—C1	104.3 (3)	H9A—C9—H9B	108.4
C2—N3—C4	128.9 (3)	C4—C10—C11	108.6 (3)
C1—N3—C4	126.8 (3)	C4—C10—H10A	110.0
N1—C1—N3	111.0 (3)	C11—C10—H10A	110.0
N1—C1—H1	124.5	C4—C10—H10B	110.0
N3—C1—H1	124.5	C11—C10—H10B	110.0
N2—C2—N3	110.3 (3)	H10A—C10—H10B	108.4
N2—C2—H2	124.8	C13—C11—C12	110.0 (3)
N3—C2—H2	124.8	C13—C11—C10	109.2 (3)
O2—C3—O1	122.1 (4)	C12—C11—C10	109.3 (3)
O2—C3—C6	119.7 (4)	C13—C11—H11	109.4
O1—C3—C6	118.2 (4)	C12—C11—H11	109.4
N3—C4—C9	109.1 (3)	C10—C11—H11	109.4
N3—C4—C10	109.1 (3)	C11—C12—C6	110.4 (3)
C9—C4—C10	110.5 (3)	C11—C12—H12A	109.6
N3—C4—C5	108.4 (3)	C6—C12—H12A	109.6
C9—C4—C5	110.2 (3)	C11—C12—H12B	109.6
C10—C4—C5	109.5 (3)	C6—C12—H12B	109.6
C4—C5—C6	109.5 (3)	H12A—C12—H12B	108.1
C4—C5—H5A	109.8	C8—C13—C11	109.2 (3)
C6—C5—H5A	109.8	C8—C13—H13A	109.8
C4—C5—H5B	109.8	C11—C13—H13A	109.8
C6—C5—H5B	109.8	C8—C13—H13B	109.8
H5A—C5—H5B	108.2	C11—C13—H13B	109.8
C7—C6—C12	108.7 (3)	H13A—C13—H13B	108.3
C7—C6—C3	112.6 (3)	O4^vi^—O4—H2W	84.2
C12—C6—C3	110.2 (3)	O4^vi^—O4—H3W	133.4
C7—C6—C5	109.1 (3)	H2W—O4—H3W	108.4
C12—C6—C5	108.1 (3)		
			
C1—N1—N2—C2	−0.1 (4)	O2—C3—C6—C5	−114.4 (4)
Ag1^iv^—N1—N2—C2	173.7 (3)	O1—C3—C6—C5	65.8 (4)
C1—N1—N2—Ag1^v^	171.1 (2)	C4—C5—C6—C7	58.0 (4)
Ag1^iv^—N1—N2—Ag1^v^	−15.1 (4)	C4—C5—C6—C12	−60.0 (4)
N2—N1—C1—N3	0.4 (4)	C4—C5—C6—C3	−179.3 (3)
Ag1^iv^—N1—C1—N3	−174.1 (2)	C12—C6—C7—C8	58.8 (4)
C2—N3—C1—N1	−0.5 (4)	C3—C6—C7—C8	−178.8 (3)
C4—N3—C1—N1	−178.4 (3)	C5—C6—C7—C8	−58.8 (4)
N1—N2—C2—N3	−0.2 (4)	C6—C7—C8—C13	−60.3 (4)
Ag1^v^—N2—C2—N3	−168.8 (3)	C6—C7—C8—C9	60.7 (4)
C1—N3—C2—N2	0.4 (4)	N3—C4—C9—C8	180.0 (3)
C4—N3—C2—N2	178.3 (3)	C10—C4—C9—C8	−60.1 (4)
Ag1—O2—C3—O1	−4.0 (4)	C5—C4—C9—C8	61.1 (4)
Ag1—O2—C3—C6	176.3 (3)	C13—C8—C9—C4	59.9 (4)
Ag1—O1—C3—O2	4.2 (4)	C7—C8—C9—C4	−61.1 (4)
Ag1—O1—C3—C6	−176.0 (3)	N3—C4—C10—C11	−179.7 (3)
C2—N3—C4—C9	170.9 (3)	C9—C4—C10—C11	60.4 (4)
C1—N3—C4—C9	−11.7 (5)	C5—C4—C10—C11	−61.2 (4)
C2—N3—C4—C10	50.1 (5)	C4—C10—C11—C13	−60.1 (4)
C1—N3—C4—C10	−132.5 (3)	C4—C10—C11—C12	60.4 (4)
C2—N3—C4—C5	−69.0 (4)	C13—C11—C12—C6	59.3 (4)
C1—N3—C4—C5	108.3 (4)	C10—C11—C12—C6	−60.6 (4)
N3—C4—C5—C6	−179.3 (3)	C7—C6—C12—C11	−58.4 (4)
C9—C4—C5—C6	−60.0 (3)	C3—C6—C12—C11	177.8 (3)
C10—C4—C5—C6	61.8 (4)	C5—C6—C12—C11	59.8 (4)
O2—C3—C6—C7	6.2 (5)	C9—C8—C13—C11	−60.8 (4)
O1—C3—C6—C7	−173.6 (3)	C7—C8—C13—C11	59.9 (4)
O2—C3—C6—C12	127.7 (4)	C12—C11—C13—C8	−59.4 (4)
O1—C3—C6—C12	−52.1 (4)	C10—C11—C13—C8	60.5 (4)

Symmetry codes: (i) −*x*+1/2, *y*−1/2, −*z*+1/2; (ii) *x*+1/2, *y*−1/2, *z*; (iii) *x*, −*y*, −*z*+1; (iv) *x*−1/2, *y*+1/2, *z*; (v) −*x*+1/2, *y*+1/2, −*z*+1/2; (vi) −*x*+1, *y*, −*z*+1/2.

### Hydrogen-bond geometry (Å, º)

**Table:**

*D*—H···*A*	*D*—H	H···*A*	*D*···*A*	*D*—H···*A*
O3—H1*W*···O1	0.85	1.97	2.818 (5)	171
O4—H2*W*···O3	0.85	1.92	2.746 (10)	163
O4—H3*W*···O5*A*^vii^	0.85	1.81	2.56 (2)	147
O4—H3*W*···O5*B*^vii^	0.85	2.11	2.83 (3)	143
C1—H1···O1^viii^	0.94	2.43	3.336 (5)	162
C2—H2···O4^vi^	0.94	2.58	3.516 (12)	171

Symmetry codes: (vi) −*x*+1, *y*, −*z*+1/2; (vii) *x*+1/2, −*y*+1/2, −*z*+1; (viii) *x*−1/2, −*y*+1/2, −*z*+1.
